# Optimal pressing strength and time for capillary refilling time

**DOI:** 10.1186/s13054-018-2295-3

**Published:** 2019-01-08

**Authors:** Rui Kawaguchi, Taka-aki Nakada, Taku Oshima, Masayoshi Shinozaki, Toshiya Nakaguchi, Hideaki Haneishi, Shigeto Oda

**Affiliations:** 1 Department of Emergency and Critical Care Medicine, Chiba University Graduate School of Medicine, 1-8-1 Inohana, Chuo, Chiba, 260-8677 Japan; 2 Chiba University, Center for Frontier Medical Engineering, 1-33, Yayoicho, Inage, Chiba, 263-8522 Japan

Substantial investigations on the microcirculation have highlighted the importance of the capillary refilling time (CRT), which is widely used in clinical settings [1–3]. However, CRT measurement conditions, including pressing strength and time, are inconsistent (e.g., pressing strength: light, moderate, or firm; pressing time: 3 s, 5 s, or until the capillary bed visually blanches) [2–5]. CRT is conventionally measured visually after manual compression, which limits the accuracy of measurement and precludes the precise determination of how long and strong the nail bed should be pressed. Therefore, we developed a novel device which can be adjusted for pressing strength and time to precisely measure CRT using an electric actuator and strength and color sensors.

CRT was measured in age- and sex-matched healthy adults (*n* = 31) using the developed device under conditions of pressing strength of 1, 3, 5, and 7 N, and a pressing time 1, 2, 3, 4, 5, and 6 s (Additional file 1: Methods and supplemental data).

There was a significant difference in CRT for pressing strength but not for pressing time (two-way analysis of variance (ANOVA): strength *P* < 0.001, time *P* = 0.97) (Fig. 1). There were significant differences in CRT between pressing strengths of 1 and 3 N (*P* < 0.01) but no significant differences among 3, 5, and 7 N (*P* = 0.16). Thus, 3–7 N appears to be the optimal strength range for CRT measurement. A strength of 3 N is that which is needed to lift an object weighing 300 g (e.g., two smartphones) using fingers; the examinees would feel a 300-g load on their fingers. To identify sufficient pressing time, we further analyzed the plots of the readings from the color sensor during nail bed compression. The measured values change rapidly in the initial part of compression (rapid phase) until the inflection point, where the slope of color change reduces (slow phase) (Fig. 2a). The longest period of the rapid phase was 1.999 s among all the study subjects (minimum 0.1022 s, 95% 1.149 s) (Fig. 2b**)**. The pressure release during the rapid phase may destabilize this measurement. Thus, a pressing time of 2 s is a good threshold to obtain stable CRT measurements for all subjects.

**Fig. 1:**
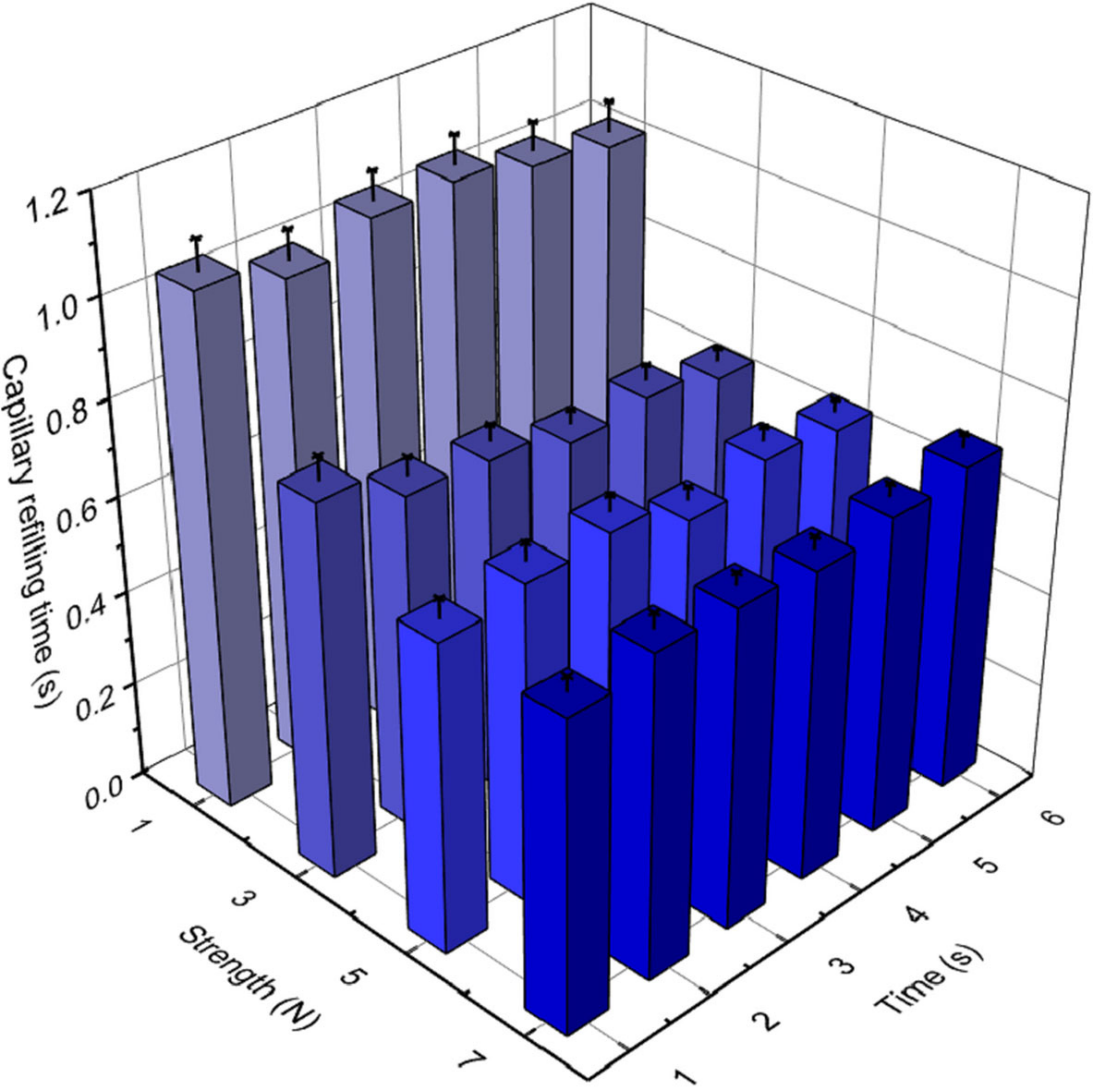
Capillary refilling time (CRT). CRT was measured in 31 age- and sex-matched healthy adults under the conditions with pressing strength 1, 3, 5, and 7 N, and pressing time 1, 2, 3, 4, 5, and 6 s in technical quintuplicate. Error bars indicate standard errors

**Fig. 2:**
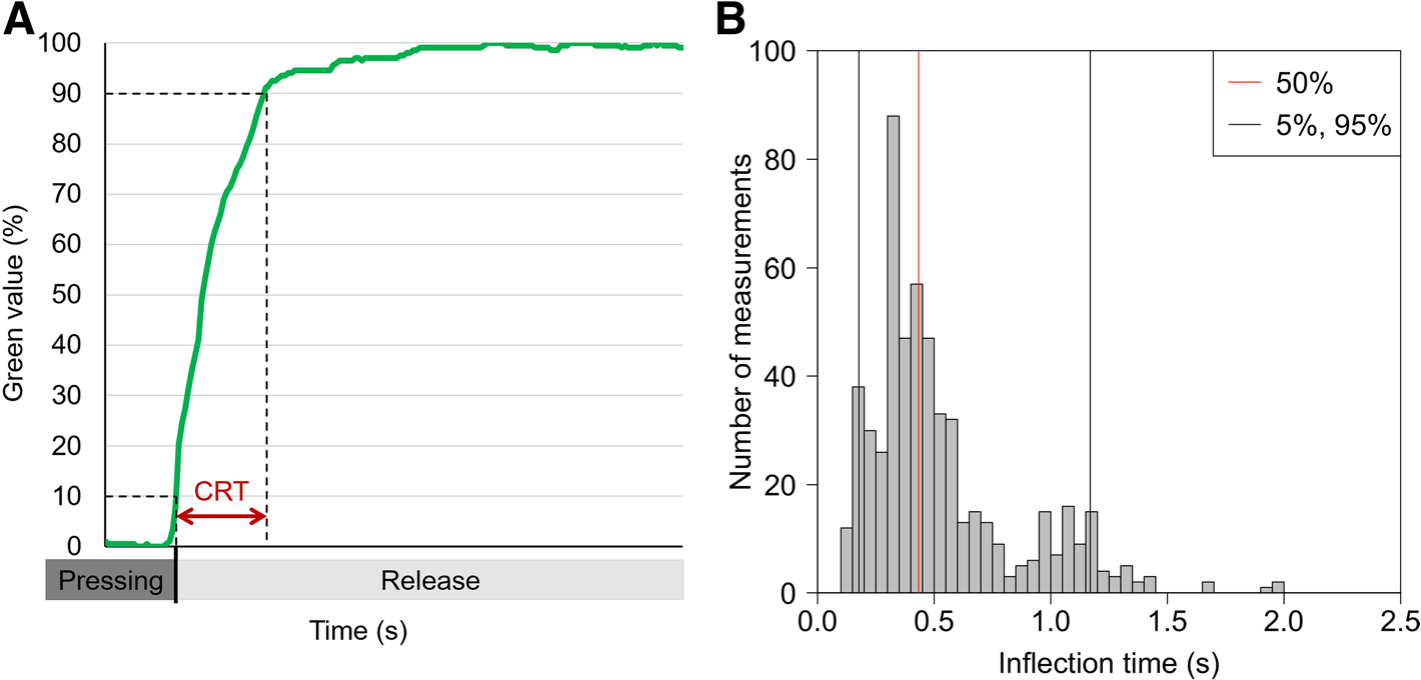
Analysis on pressing time. **a** A schema of curve plot of the readings by the color sensor. The value sensing by color sensor rapidly changes in the initial part of the pressing nail bed (rapid phase) until the inflection point and then the slope of color change become slow (slow phase). **b** Histogram of inflection times (*n* = 744 (31 subjects × 24 conditions)). Gray lines indicate 5% and 95% lines. The red line indicates 50% time line. CRT capillary refilling time

In the study aimed at identifying the standard pressing strength and CRT, pressing the nail bed with 3–7 N for 2 s appears to be optimal. Further development of portable CRT measurement devices fulfilling these conditions may contribute to achieving precise CRT measurements to monitor the microcirculation in clinical settings.

## Additional file

Additional file 1: Methods and supplemental data. **Figure E1.** Picture of developed device. The upper image is the uncovered condition to show the internal structure. The lower image is the covered condition in which we measured CRT of study subjects. 1) Stepper motor. 2) Uni-axial stage. 3) Color sensor, light source and indenter. 5) Industrial camera. 6) Emergency stop switch. 7) Cover for hiding press principle. Size 16 cm × 20 cm × 23 cm. **Figure E2.** Principle of the device. **Figure E3.** Definition of CRT. **Table E1.** Characteristics of subjects and data. (DOCX 29524 kb)
