# Supplementary material for: Optimal pressing strength and time for capillary refilling time
Source: Crit Care. 2019 Jan 8;23:4. doi: 10.1186/s13054-018-2295-3 (PMC6323707; doi:10.1186/s13054-018-2295-3)
Supplement: Supplementary file 1 — Methods and supplemental data. Figure E1. Picture of developed device. The upper image is the uncovered condition to show the internal structure. The lower image is the covered condition in which we measured CRT of study subjects. 1) Stepper motor. 2) Uni-axial stage. 3) Color sensor, light source and indenter. 5) Industrial camera. 6) Emergency stop switch. 7) Cover for hiding press principle. Size 16 cm × 20 cm × 23 cm. Figure E2. Principle of the device. Figure E3. Definition of CRT. Table E1. Characteristics of subjects and data. (DOCX 29524 kb) [file 13054_2018_2295_MOESM1_ESM.docx]

**Optimal pressing strength and time for capillary refilling time**

Rui Kawaguchi, Taka-aki Nakada, Taku Oshima, Masayoshi Shinozaki, Toshiya Nakaguchi, Hideaki Haneishi, Shigeto Oda

***Online data Supplement***

**1. Methods**

**1.1 Device**

We developed a novel device which consists of (1) a finger nail bed pressing system that enables to precisely push the nail bed with a specific strength for a specific time, and (2) a measurement system to detect the color change of the nail bed (**Figure E1**). The finger nail bed pressing system is made using a stepper motor and a uniaxial stage (**Figure E2**). The rotation of the stepper motor is transformed to an upward and downward movement of the stage, allowing the press and release of the nail bed. The shifting of the stage is controlled using the rotation angle of the stepper motor (NEMA17 JK42HS34-1334 (Step angle: 1.8°, Current/Phase: 1.33 A, Holding torque: 2.6 kg·cm). A load cell (KYOWA, LMA-A-50N, Japan) can precisely control the pressure, and a timer from a microcomputer can control the time for pressing the nail bed. The color change was measured by a reflect type photosensor—a color sensor (Adafruit Industries, RGB Color Sensor with IR filter and White LED – TCS34725, USA, integration time: 24 ms, quadruple gain), and the light source illumination was provided by a white LED. To monitor the condition of the fingertip during the measurement, we captured images from the side with an industrial color camera (Imaging source, DFK22BUC03).

**1.2 Definition of the capillary refilling time** (**CRT)**

CRT was calculated from the data collected from the color sensor. The G component waveform had the biggest change among the R, G, and B components. Thus, we chose the G component of the color for analysis in this study. The color of the nail bed when it was compressed was regarded as 100%, and the color after the nail bed was released and stabilized was regarded as 0%. The CRT was defined as the time taken for the color to return from 90% to 10% (**Figure E3**).

**1.3 Subjects and Study Protocol of Measurements**

A total of 31 healthy adults were studied (**Table E1**). CRT of the right index finger of the subjects were measured using the developed device. CRT was measured under the condition with pressing strength 1, 3, 5, and 7 N, and pressing time 1, 2, 3, 4, 5, and 6 s. Among each of the 24 conditions, CRT measurements was repeated 5 times and the averaged value of 5 measurements was used for the analysis. Thus, we collected 744 CRT values in total (31 subjects × 24 condition). The measurement was performed starting from the condition of 1 N and 1 s and repeated 5 times with 3 s interval. We also measured thickness of the finger by a Vernier caliper and fingertip temperature using a contact-less infrared thermometer before and after CRT measurements.

**1.4 Statistical Analysis**

We first analyzed using two-way ANOVA for the 24 conditions (4 strength conditions and 6 time conditions). We compared CRTs between 1 N and 3 N using the t-test and among 3, 5, and 7 N using one-way ANOVA. All of the statistical analyses were performed using R software (version 3.5.0.).

**Table E1. Characteristics of subjects and data**

|  | Male | Female |
| --- | --- | --- |
|  | (n=14) | (n=17) |
| Age -n |  |  |
| 20 – 29 y | 3 | 4 |
| 30 – 39 y | 3 | 2 |
| 40 – 49 y | 3 | 4 |
| 50 – 59 y | 2 | 5 |
| 60 – 69 y | 2 | 1 |
| 70 – 79 y | 1 | 1 |
| Thickness of the finger -mm |  |  |
| 20 – 29 y | 11.68±0.68 | 11.77±1.30 |
| 30 – 39 y | 11.20±1.28 | 10.40±0.30 |
| 40 – 49 y | 11.47±0.21 | 12.02±1.20 |
| 50 – 59 y | 10.92±0.62 | 12.31±1.49 |
| 60 – 69 y | 10.26±1.29 | 13.00 |
| 70 – 79 y | 11.85 | 12.00 |
| Fingertip temperature |  |  |
| Before measurement -℃ |  |  |
| 20 – 29 y | 31.4±3.2 | 32.8±0.4 |
| 30 – 39 y | 33.2±0.4 | 31.5±1.4 |
| 40 – 49 y | 32.3±0.5 | 31.9±0.7 |
| 50 – 59 y | 26.8±1.0 | 31.4±3.6 |
| 60 – 69 y | 31.7±2.2 | 30.3 |
| 70 – 79 y | 29.4 | 34.2 |
| After measurement -℃ |  |  |
| 20 – 29 y | 32.4±2.0 | 32.7±0.7 |
| 30 – 39 y | 33.2±0.7 | 30.8±0.2 |
| 40 – 49 y | 32.9±0.7 | 32.9±1.2 |
| 50 – 59 y | 29.8±2.8 | 32.4±1.9 |
| 60 – 69 y | 28.2±2.1 | 30.6 |
| 70 – 79 y | 33.7 | 33.6 |

Data are mean±SD.

**Figures Legend**

**Figure E1. Picture of developed device**

Upper image is an uncovered condition to show the internal structure. Lower image is a covered condition in which we measured CRT of study subjects.

1 Stepper motor

2 Uni-axial stage

3 Load cell

4 Color sensor, light source and indenter

5 Industrial camera

6 Emergency stop switch

7 Cover for hiding press principle

Size 16 cm x 20 cm x 23 cm

**Figure E2. Principle of the device.**

**Figure E3. Definition of CRT. Figure E1. Picture of developed device**

**
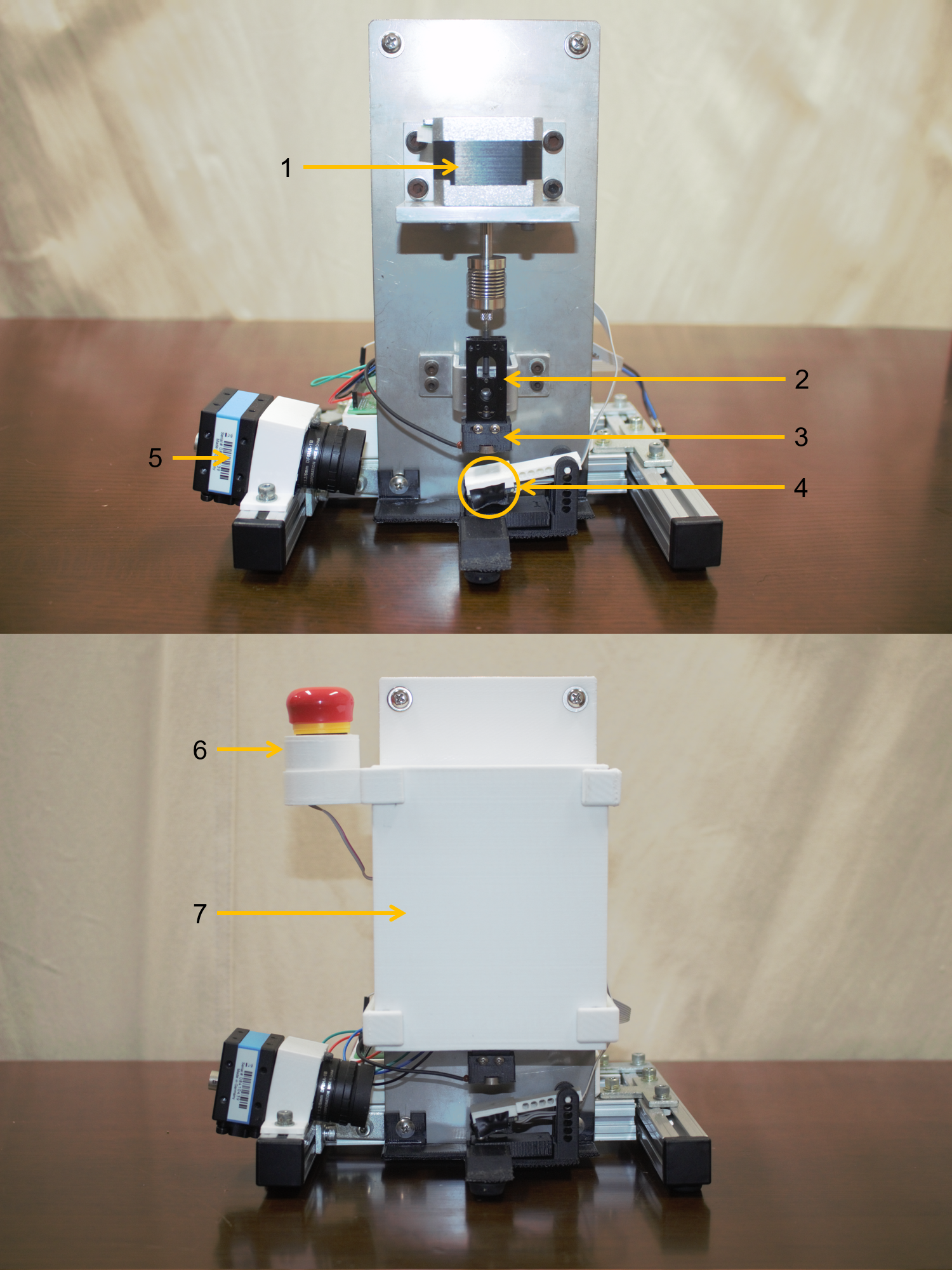
**

**Figure E2.**

**
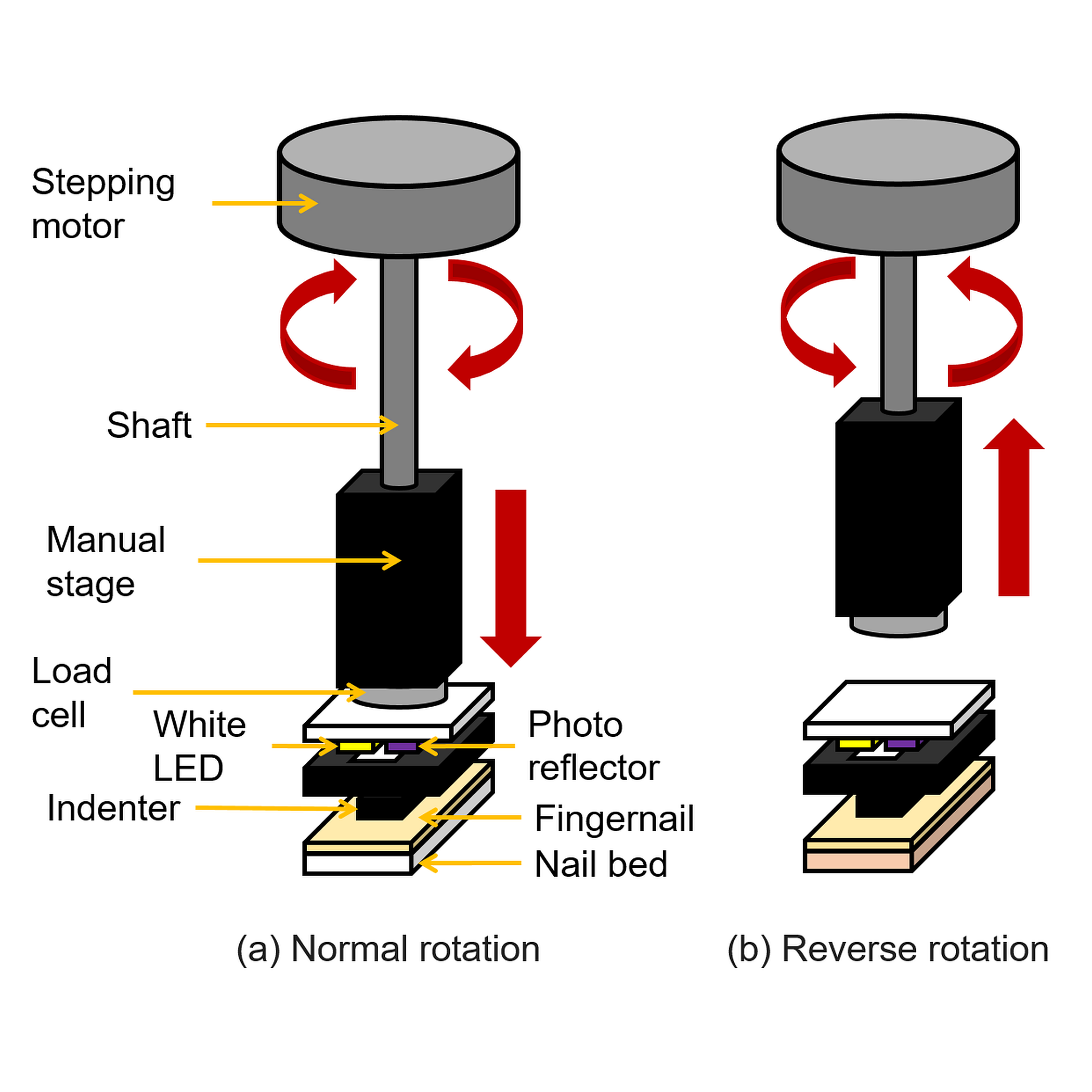
**

**Figure E3. Definition of CRT**

**
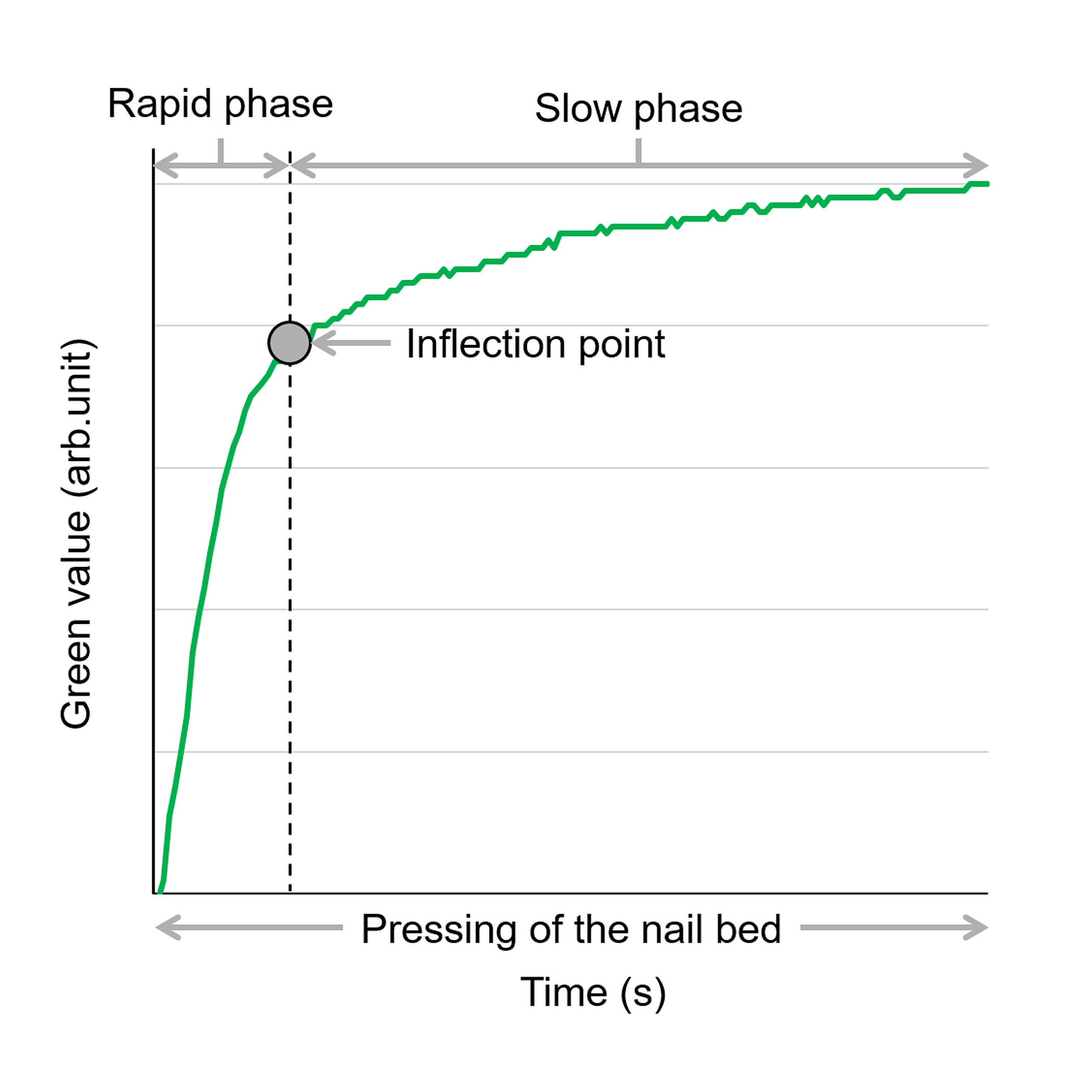
**
